# Multi-silicone bilateral soft physical twin as an alternative to traditional user interfaces for remote palpation: a comparative study

**DOI:** 10.1038/s41598-023-50329-4

**Published:** 2023-12-27

**Authors:** Leone Costi, Fumiya Iida

**Affiliations:** Bio Inspired Robotics Laboratory, Department of Engineering, University of Cambridge, Cambridge, UK

**Keywords:** Energy science and technology, Engineering, Health care

## Abstract

Teleoperated medical technologies are a fundamental part of the healthcare system. From telemedicine to remote surgery, they allow remote diagnosis and treatment. However, the absence of any interface able to effectively reproduce the sense of touch and interaction with the patient prevents the implementation of teleoperated systems for primary care examinations, such as palpation. In this paper, we propose the first reported case of a soft robotic bilateral physical twin for remote palpation. By creating an entirely soft interface that can be used both to control the robot and receive feedback, the proposed device allows the user to achieve remote palpation by simply palpating the soft physical twin. This is achieved through a compact design showcasing 9 pneumatic chambers and exploiting multi-silicone casting to minimize cross-noise and allow teleoperation. A comparative study has been run against a traditional setup, and both the control and feedback of the physical twin are carefully analyzed. Despite distributed tactile feedback not achieving the same performance as the visual map, the soft control and visual feedback combination showcases a 5.1% higher accuracy. Moreover, the bilateral soft physical twin results always in a less invasive procedure, with 41% lower mechanical work exchanged with the remote phantom.

## Introduction

Palpation is one of four phases of physical examination, the most common routine test performed by general practitioners in order to assess the presence of abnormal tissues or nodules in the patient’s abdomen^1^. With the only requirement of the doctors’ hands and expertise, palpation can help in both selecting the most suitable follow-up exams and finding the correct diagnosis^2^. For such reasons, physical examination represents a fundamental step in today’s healthcare system, and when performed correctly can strongly reduce times and costs both for the patient and the healthcare system. Among its 4 phases (inspection, auscultation, palpation, and percussion), palpation, due to its prolonged mechanical interaction between the practitioner’s hand and the patient’s abdomen, has been the focus of several quantitative studies, both in simulation^3^ and in clinical scenarios^4^. Naturally, palpation requires the practitioner and patient to be physically present in the same room. However, due to a multitude of factors, such as a global pandemic or the impossibility of the doctor reaching the patient’s location (e.g. space applications), it is not always possible to perform in-person palpation. Teleoperation and remote diagnosis offer a potential solution, by allowing doctors to perform the examination from the hospital in any remote environment. This can potentially lead to both the diagnosis of unreachable patients and the minimization of the drawbacks produced by the ever-growing shortage of physicians^5^.

Teleoperated robotic systems are human-in-the-loop systems that allow the remote execution of a task^6^. They are characterized by a leader, which is the interface used by the user to control the robot, and a follower, which is the robot in the remote environment performing the action. Medical practice has always been an ideal field of application due to the time-sensitive nature of medical examinations: the possibility of having a skilled user (e.g. a general practitioner) operating remotely in any arbitrary location can potentially lead to faster and better diagnosis, both improving the life quality of the patient and reducing costs^7^. For such reasons, teleoperated systems have already permeated the medical and surgical fields, from minimally invasive surgery to endoscopy^8,9^. However, when considering the task of palpation, distributed tactile haptic feedback is of fundamental importance^10^ and makes such a task a great topic to study robotic soft body interaction^11–13^. Conversely to commercial teleoperated surgical robots^14–17^ and kinesthetic haptic feedback interfaces present in literature^18–21^, robotic systems for remote palpation require distributed tactile feedback^22^. Moreover, the leader needs to be a bilateral interface and deliver distributed tactile haptic feedback while allowing the user to control the follower.

Most of the studies in the literature are centered around the different ways to deliver tactile data back to the user. Transparency and intuitiveness, where intuitiveness means achieving telepresence with minimal learning, are fundamental for achieving good teleoperation and telepresence^23,24^. The simplest systems just show the data on a screen either by maintaining the spatial information recorded by the follower^25,26^, or through simulations^27–29^. By effectively creating a unilateral architecture, these approaches can be implemented in commercial devices without the risk of instabilities, but deliver the tactile data visually rather than haptically, thus resulting in less natural and intuitive interfaces. Another possible solution is vibrating stimulation devices to deliver haptic feedback. They can be placed on the user’s finger^30^ or over the control interface^31^. These devices are very compact and can easily be used with pre-existing systems. However, they do not retain the exact spatial information of the stimulus and can only encode its magnitude, thus they are not suitable for distributed tactile feedback. The most advanced interfaces are physical twins: devices that can change their morphology in order to mimic the surface touched by the follower, acting as an identical copy of the remote environment. Such devices can achieve the required morphology changes using traditional components such as servomotors and rigid components^32,33^, or can use soft actuation for a more compact design and a closer resemblance to the human skin^34^. They are able to convey distributed tactile haptic feedback and can be used as a part of the leader.

Nevertheless, the leader is not only a feedback interface but is also used to control the follower. On one hand, this can be achieved by a physical twin in conjunction with a traditional control interface. However, it would require the user to split the attention between controlling and analyzing the feedback, potentially resulting in a loss of performance. On the other hand, the physical twin, if properly designed, can be used also as a control interface, creating a bilateral system. Bilateral physical twins can also be made with traditional components^35,36^, or by using soft robots^37^. However, all reported cases of bilateral physical twins either have extremely limited degrees of freedom (DoFs) or have solely been tested in simulation and not in a real leader-follower architecture. Recently, Costi et al.^38^, have demonstrated how it is possible to use barometric sensors in a silicone interface to achieve precise dexterous control of up to 5 DoFs.

**Figure 1:**
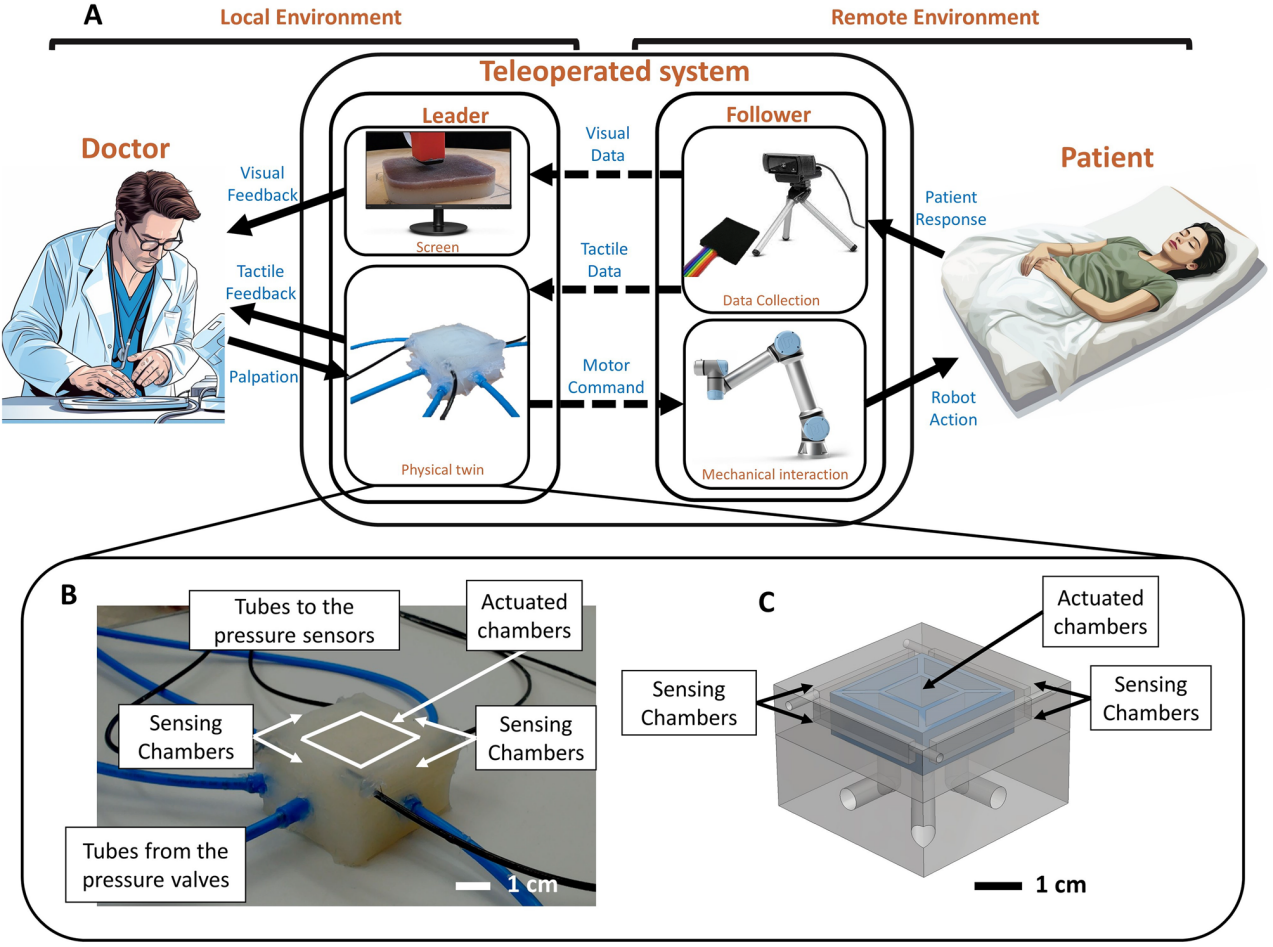
(**A**) Schematics of a bilateral teleoperated system for remote palpation with (**B**) picture and (**C**) 3D model of the proposed physical twin. The system consists of four parts: the practitioner, the leader, the follower, and the patient. The practitioner interacts directly with the physical twin to control the motion of the follower. The control interface only requires the practitioner to palpate the physical twin as if it were the patient in the case of in-person palpation. The motor command is then encoded by the leader and the robotic arm executes the trajectory, touching the patient. The tactile data about the patient’s abdominal tissues is then collected with a tactile sensor, while a camera films the interaction. The camera feed is transmitted in real-time to the practitioner through a screen, whereas the tactile data is transferred to the physical twin. The physical twin then closes the haptic loop by delivering distributed tactile haptic feedback to the practitioner.

In this work, we propose an interface for remote palpation without adapting tactile haptic feedback on an already existing teleoperated system, but we exploit soft robotics and pneumatic technologies to develop the first reported case of a physical twin for remote palpation. Figure 1A shows the overall architecture, as well as the role that the bilateral physical twin plays both in the control of the follower and in the distributed tactile feedback delivery. Figure 1B illustrates a close-up of the soft physical twin, and Fig. 1C reports the relative 3D model. The proposed interface has the aim to effectively reproduce the sense of touch remotely, closing the gap between remote palpation and in-person palpation. To do so we make large use of soft robotics by combining previous design principles about soft sensing^38^ and soft actuation^34^ to obtain an entirely soft bilateral device with the attempt to resemble as close as possible the intrinsic compliance characteristic of the hand-abdomen contact. Moreover, we utilize the design principle of multi-silicone casting to ensure high sensitivity in the sensing chambers and reduce cross-noise and potential actuation-driven instabilities.

To quantify and fully characterize the performance of the soft bilateral physical twin, a comparative study has been performed, in which participants are asked to correctly identify the location of a hard nodule within a silicone phantom. To isolate the performance of both the tactile haptic feedback and the soft control interface, all participants are tested in six different conditions, covering all possible combinations of traditional or soft control and tactile or visual feedback. Examples of the users under the 6 tested conditions are shown in Movie S1. The results highlight how the soft bilateral physical twin allows the users to apply significantly less force to the remote phantom when performing the task. Moreover, the soft physical twin is also shown to be a better control interface than the traditional keyboard, with a 5.1% higher diagnostic accuracy.

In the remainder of the paper, section “Results” reports the results obtained in the comparative study, followed by the discussion in section “Discussion” and the design and manufacturing of the device and the system, as well as the description of the experimental protocol in section “Materials and methods”.

## Results

**Figure 2:**
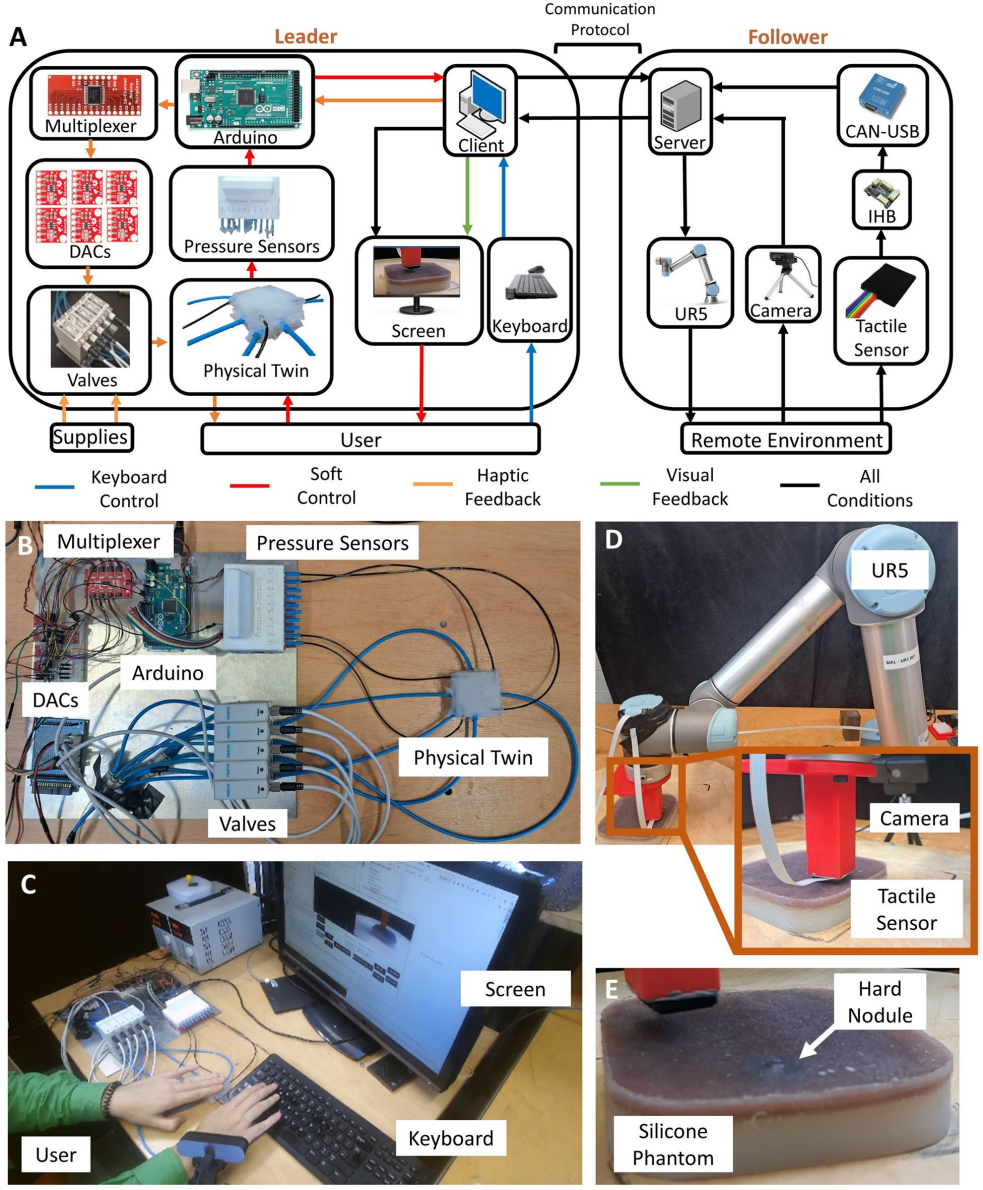
(**A**) Overall schematics of the teleoperated system. The visual tactile map and the tactile haptic feedback can be delivered individually or together, and either the keyboard or the physical twin can be used for motion control. Pictures of (**B**) the physical twin and the leader’s hardware needed to use it, (**C**) the user interacting with the leader during a task, (**D**) the robotic arm acting on the silicone phantom in the remote environment, and (**E**) a close-up view of the silicone phantom and the nodule placement used in the experimental protocol.

This paper proposes a soft bilateral physical twin as an interface in a teleoperated system for remote palpation. To analyze the performance of such a device, a comparative study is performed, in which both the physical twin and a combination of keyboard and visual pressure map are used in a remote palpation localization task. Figure 2A illustrates the system that has been implemented for the study. The system is composed of a leader and a follower which communicate through User Datagram Protocol (UDP). The leader can be tuned to either make use of the motor commands delivered by the keyboard or by the physical twin, as well as delivering the tactile data visually or through the distributed tactile haptic feedback of the physical twin. In all configurations, the user is also able to see a real-time camera feed of the remote environment. Note that considering all the possible combinations of control and feedback, six different configurations are investigated in the comparative test: keyboard with visual feedback, keyboard with haptic feedback, keyboard with both visual and haptic feedback, soft control with visual feedback, soft control with haptic feedback, soft control with both visual and haptic feedback. Soft control indicates that the physical twin, and not the keyboard, is being used to control the follower. A detailed description of the different conditions is provided in section “Materials and methods”. Figure 2B and C show how the physical twin has been implemented within the leader and how the user is able to interact with the leader itself, respectively. Conversely, Fig. 2D and E show the remote environment, and how the robotic arm interacts and collects data, through the tactile sensor, from the silicone phantom.

**Figure 3:**
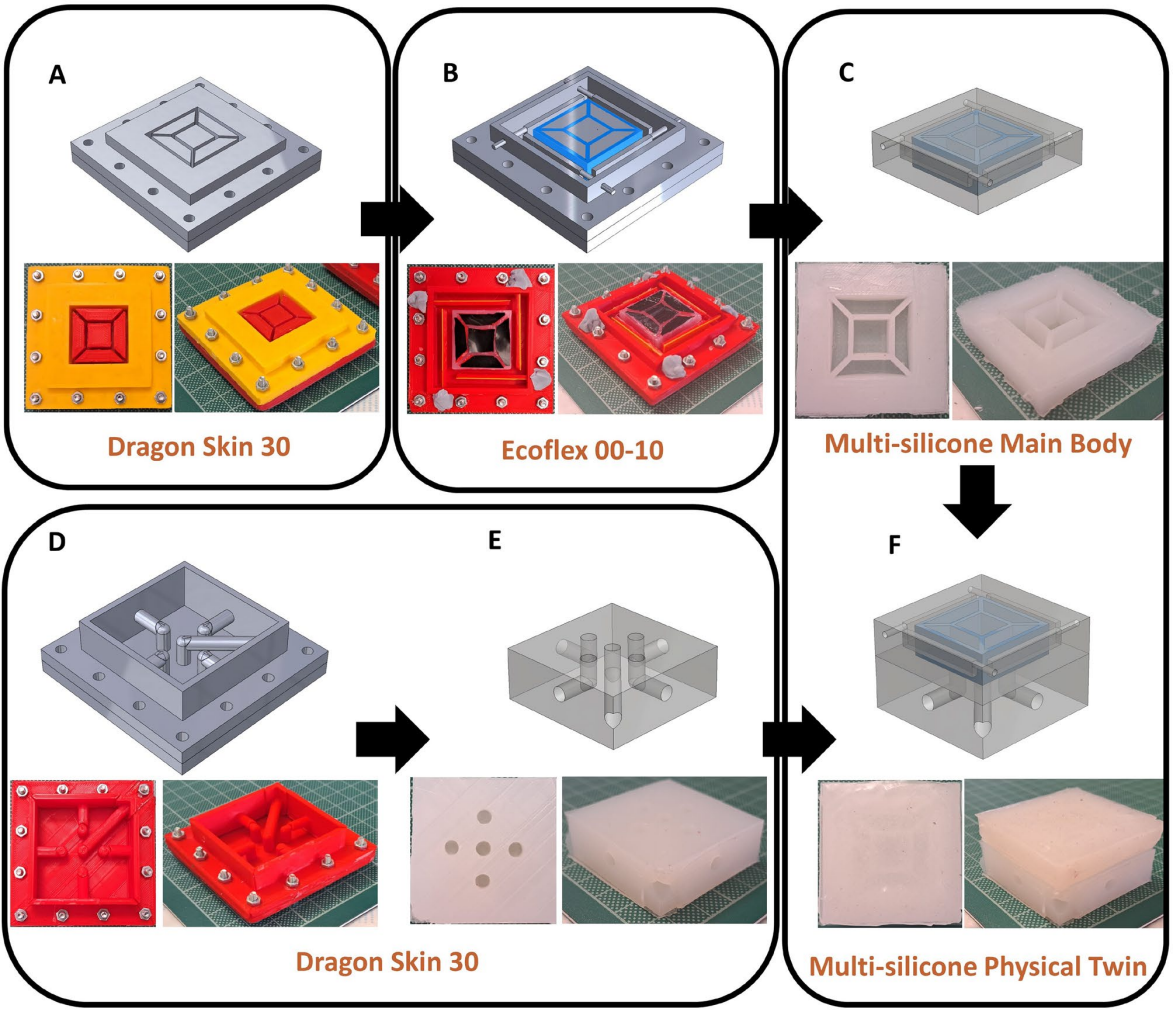
3D models and top and side pictures of the manufacturing process of the soft physical twin. The main body of the device is fabricated by (**A**) silicone-casting Dragon Skin 30 in the mold for the inner core, (**B**) silicone-casting Ecoflex 00-10 in the mold for the complete main body, (**C**) extracting the main body from the mold, (**D**) silicone-casting Dragon Skin 30 in the mold for the base, (**E**) extracting the base from the mold, and (**F**) assembling the physical twin.

Concerning the design of the interface, it is fundamental to localize the deformation toward the user’s hand and minimize cross-noise between sensing and actuated chambers. Hence, the physical twin is obtained with a multi-silicone casting in 2 steps (see Fig. 3). First, a Dragon Skin 30 core is cast: this relatively stiff material is used to manufacture the walls of the actuated chambers to minimize the displacement of the side walls. Then, using silicone glue to achieve a robust interface, the remainder of the physical twin is cast using Ecoflex 00-10: this comprehends both the top side of the actuated chambers, in contact with the user’s hands, and the sensing chambers. Further details about the assembling of the molds are reported in Fig. S1. By multi-silicone-casting two materials that largely differ in mechanical properties, the structural deformation is directed toward the Ecoflex 00-10 parts. On one hand, this results in augmenting the sensitivity of the sensing chambers and the tactile haptic feedback delivered to the user. On the other hand, the inner Dragon Skin 30 core undergoes limited deformation, minimizing the cross-noise that could be created by the actuation of the five inner chambers onto the sensing ones, laterally placed. In turn, this allows the implementation of a much more compact design, allowing sensing and actuating chambers to be relatively close. Dragon Skin 30 is also used to cast the base of the interface, which contains the connections pneumatic connections used to pressurize the actuated chambers. In other words, the selected design exploits both multi-silicone deformation directionality and morphological computation to achieve the encoding of the user’s desired trajectory and delivery of the tactile feedback simultaneously. Further details about the manufacturing process are described in section “Materials and methods”.

**Figure 4:**
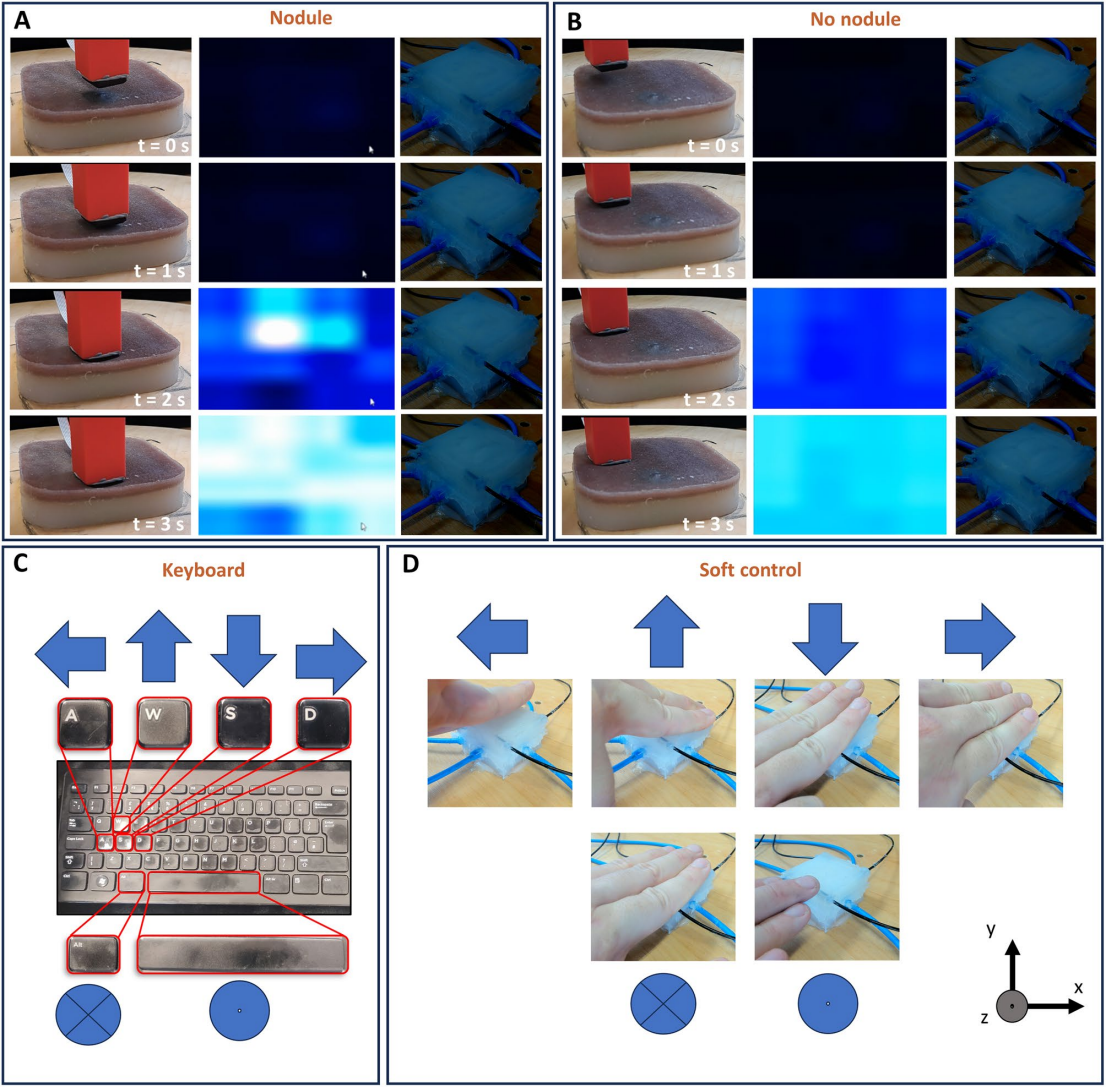
Feedback signals available to the users when palpating (**A**) a nodule or (**B**) the plain soft silicone phantom. The real-time camera feed to see the robot’s position is on the left, whereas the haptic data is delivered as a visual map and/or haptic feedback, in the center and on the right, respectively. Control modalities to move the follower: (**C**) keyboard or (**D**) soft physical twin. The soft physical twin utilizes the 4 sensing chambers to allow the user to control the follower by palpating the physical twin itself: shifting the hand’s pressure toward a direction would move the robot in such direction, and pressing down uniformly would make the follower palpate the phantom.

While performing remote palpation with the developed system, users have access to the visual representation of the tactile data and/or the tactile haptic feedback (see Fig. 4A and B), depending of the tested condition. The visual representation maps high-pressure values to white and low-pressure ones to black, following a blue scale, whereas the physical twin maps pressure values directly to chamber pressure. However, the physical twin has only 5 actuated chambers, so the 16 pressure values recorded by the tactile sensor are grouped according to their spatial location. Because the tactile sensor is much larger than the simulated nodule, when palpated it would produce a not uniform pressure distribution. Conversely, palpating plain silicone results in a much more uniform one. Moreover, the follower can be controlled with the keyboard or using the soft physical twin (see Fig. 4C and D). Using the keyboard, the user is able to control the follower simply using 6 keys, as previously implemented in literature^39^. Conversely, when using the soft physical twin as a control interface, the user can move the follower by palpating the physical twin itself. An in-depth description of how the physical twin’s pressure signals are turned into motor commands for the follower is provided in section “Materials and methods”.

**Figure 5:**
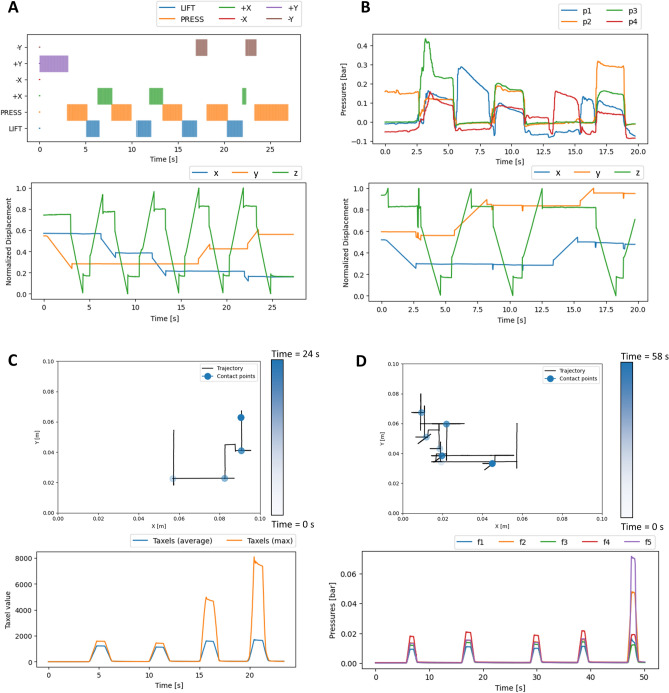
Examples of control and feedback in the different conditions: (**A**) keyboard control inputs and achieved trajectory as functions of time, (**B**) soft control inputs and achieved trajectory as functions of time, (**C**) visual feedback as a function of time and relative trajectory, (**D**) tactile haptic feedback as a function of time and relative trajectory. In the feedback trajectory plots, the intensity of the marker represents the time: lighter points have been palpated at the beginning of the trial, whereas darker ones have been palpated toward the end. *p* is used to indicate the pressure in the sensing chambers, whereas *f* is used for the actuated chambers. The chamber’s index represents the specific chamber.

To further analyze such conditions, Fig. 5 shows examples of control and feedback modalities accessible by the user in the different conditions, as well as the achieved kinematics in the remote environment. Firstly, it can be seen how the inputs relate to the motion of the robot. In the case of keyboard input, key presses result in a constant velocity motion toward that direction. Motion along multiple axes is possible by simultaneously pressing multiple keys. In the case of soft control, the follower is moved by increasing the pressure of the sensing chamber in the intended direction. Because of the placement of the chambers, this is achieved simply by moving the hand toward the desired side. Then, palpation is achieved by pressing uniformly on the physical twin itself, as can be seen by the follower pressing down along *z* when all sensed pressures are above a given threshold and lifting off otherwise. It can be seen in the normalized displacement plot in Fig. 5B that at times there can be unwanted jerking motions along *x* and *y*, indicated by small rapid deviations. These unwanted oscillations usually happen as the user is pressing on the phantom (e.g. descending *z*) and are caused by the transients of the recorded pressure (e.g. as the user starts pressing or lifting the hand). Nevertheless, they represent relatively small displacements with respect to the overall trajectory. Concerning the visual and haptic feedbacks (see Fig. 5C and D, respectively), the trajectories show how the user explores the remote environment. While trying to localize the nodule in the shortest possible time, it is common that not all of the surface is explored, but rather an educated guess is made based on partial information. Lastly , the feedback signal shows how the information about the presence or absence of the nodule is shown to the user. When relying on the visual map, the presence of the nodule under the tactile sensor nodule will produce pressure peaks (See Fig. 4A). This can be observed in the difference between the average and maximum taxel values displayed. Similarly, when using the distributed tactile haptic interface, the presence of the nodule can be inferred by correctly identifying that one of the chambers showcases a much higher pressure, thus delivering stronger feedback to the user’s hand. More examples of control and feedback as a function of the testing conditions are provided in Fig. S3.

**Figure 6:**
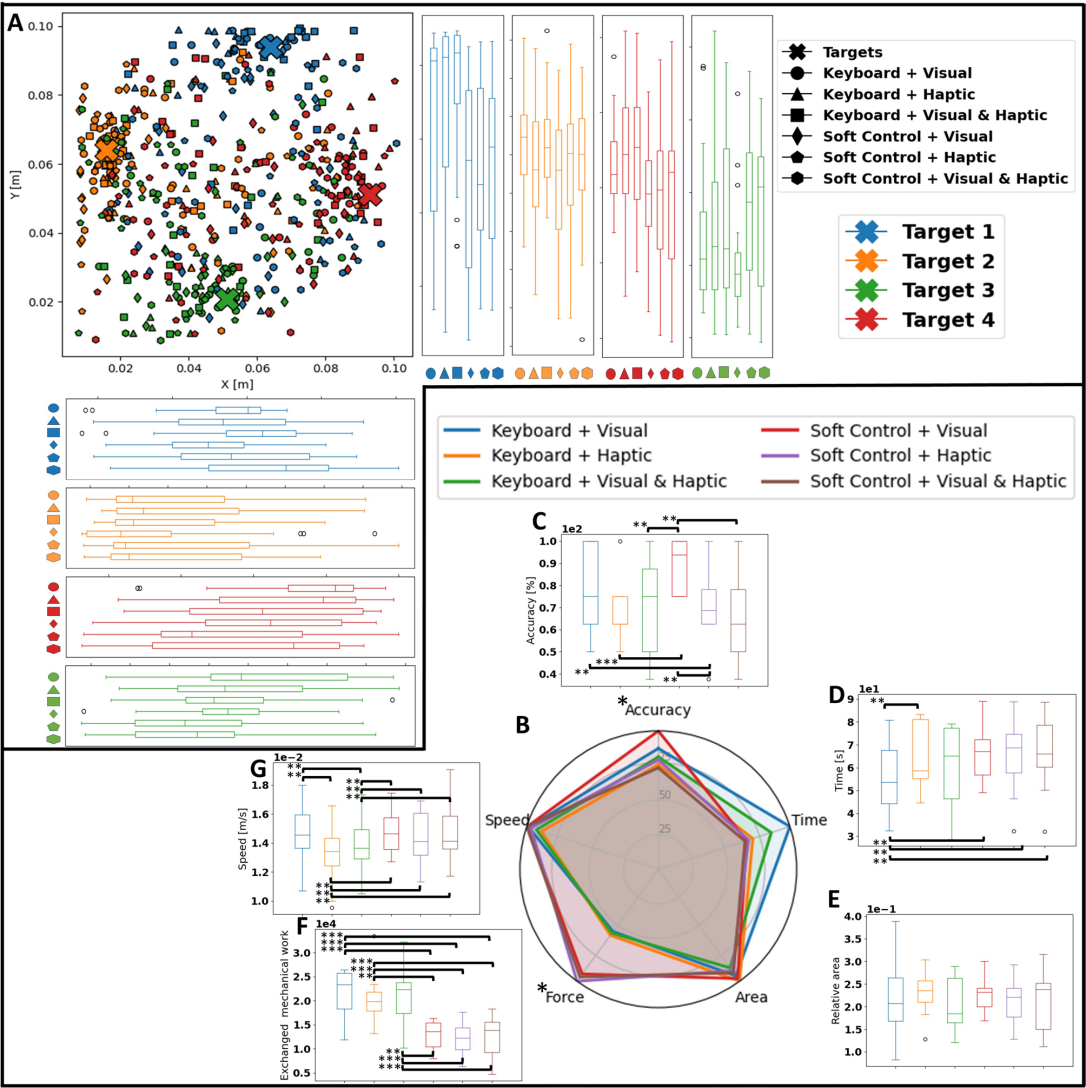
(**A**) Users’ estimations of nodules location as a function of nodule’s true location and testing conditions. Note that the target location is indicated by the color of each marker, with the true location indicated by the bigger cross-shaped marker, whereas the testing conditions are indicated by its shape, according to the legend. (**B**) Radar chart and (**C**–**G**) box plots of the 5 selected metrics: (**C**) accuracy, (**D**) time, (**E**) area, (**F**) force, and (**G**) speed. To highlight statistical significance one-way ANOVA has been run for each metric and paired t-tests have been run for every combination of conditions: $$*$$ represents statistical significance in the ANOVA, whereas $$**$$ and $$***$$ represent $$p<0.05$$ and $$p<0.001$$ in the paired t-test, respectively. Note that the colors in (**B**–**D**) represent the testing conditions, as indicated in the legend. Moreover, the metrics in the radar chart have been adjusted so that higher values always correspond to better performance, and have been scaled with respect to the maximum score across modalities..

Concerning the group study, Fig.  6 and Table 1 report all the group study results. Moreover, the different conditions have been evaluated using five metrics extracted from the users’ performance: accuracy, time, speed, area, and force. Accuracy is the percentage of trials in which the nodule’s position has been identified correctly, time is the trial’s time, speed is the follower’s average speed maintained during the trial, area is the percentage of the phantom’s surface that has been explored, and force is the overall mechanical work exchanged with the remote environment. Further details of how these metrics have been computed in every individual trial are provided in section “Trial’s metrics”, as well as the data processing needed for the radar chart and the statistical tests in sections “Radar chart” and “Statistical tests”, respectively.

From Fig. 6A, it can be seen how different conditions affect the users’ ability to correctly localize the nodule’s position. In most cases, the two modalities relying solely on the visual pressure map achieve more dense clusters around the ground truth. Conversely, when using only tactile haptic feedback, users are more prone to errors. This can be attributed both to the lower spatial resolution since the physical has only 5 actuated chambers and to the novelty of the physical twin as a feedback interface, to which the users need to adapt. This phenomenon is especially noticeable in the closest and further target locations to the user (green and blue, respectively). However, when using both feedback modalities, the spatial distribution of the users’ answers is not as well clustered as when using only visual feedback, and it is very similar to the one of the sole tactile haptic feedback. This indicates how overwhelming the users with all possible information prevents them from correctly interpreting the tactile data and lowers their performance. Fig. 6B shows an overall characterization of the six different conditions, which can be further analyzed in Fig. 6C–G. The interface change has been shown to be statistically significant for the accuracy and the force when tested with a one-way ANOVA, as shown in Table 1. Further details on the datasets used in the test are provided in section “Statistical tests”. Moreover, the normality of the datasets has been investigated using a Shapiro-Wilk test and a further in-depth comparison between the 6 conditions for each metric has been carried out using a paired t-test on any possible condition combination (see Tables S1 and  S2- S6, respectively). Once again, it can be seen how visual feedback achieves the highest accuracy, while a combination of visual and tactile performs worse than either feedback individually. In fact, soft control combined with visual feedback is proven to achieve higher accuracy than traditional visual feedback and keyboard. Fig. 6C also confirms that overwhelming the users with multiple feedback signals does not improve the accuracy, but rather lowers it even more than both of the single feedback conditions. To further investigate the reasons behind the change in accuracy, both explored area and exploring speed are analyzed (Fig. 6E and G, respectively). Concerning the explored area, all tested conditions resulted in a similar performance, with no statistically significant difference. Conversely, the average speed achieved using soft control matches the one of traditional control, regardless of the feedback modality, while keyboard control results in a significantly slower motion when combined with haptic feedback. Moreover, since the instantaneous speed of the end-effector is constant in order to compare the different interfaces, it can be inferred that users tend to spend more time not moving while controlling the robot with the keyboard when compared to the physical twin. Lastly, the introduction of soft control, especially when combined with sole tactile haptic feedback, achieves a much lower force exchanged with the remote environment (see Fig 6F), resulting in a much less invasive examination, as statistically proven both by the ANOVA and paired t-tests. However, any variation from the traditional interface, with the exception of the keyboard and both feedbacks, results in a statistically significant increment in the required time (see Table S3), probably due to the longer training process needed for interfaces that are extremely novel for the users. User-specific metric results are provided in Fig. S4.

**Table 1:** One-way ANOVA results.

	Accuracy	Time	Area	Force	Speed
F-value	3.186	1.051	0.365	11.213	1.466
p-value	0.012	0.396	0.871	$<0.001$	0.213

Overall, the soft physical twin is shown to achieve similar abilities of surface exploration, while allowing a much less invasive examination. Noticeably, using the physical twin as a control interface leads to faster trajectories and higher diagnostic accuracy. However, the introduction of novel methods of control and feedback results in slower examination times, and the distributed tactile haptic feedback, both alone or with the visual pressure map, is not able to match the accuracy obtained with the map itself. On one side, this can be due to the lower spatial resolution of the tactile feedback, which showcases only five chambers instead of the 16 taxels of the visual map. On the other hand, the reported results are strongly affected by the learning curve of the participants. Differently from keyboard and visual feedback, both the soft control and the tactile haptic feedback are interfaces that participants have never experienced before. This results in the users improving their performance over time regardless of the order in which they try the different conditions. All in all, despite the tactile haptic feedback not matching the performance that users are able to obtain with the visual map, the soft control is proven to be extremely effective, with a statistically significant increased accuracy and lowered invasiveness, while maintaining similar speed and explored area, and slightly longer time.

**Figure 7:**
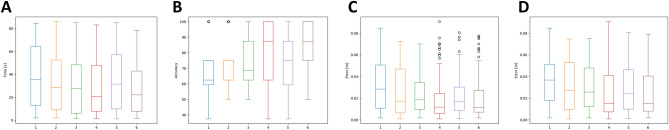
Learning curves in the case of (**A**) time needed to localize the nodule after the first contact was made, (**B**) accuracy, (**C**) error when the tumor was correctly localized, and (**D**) general error.

To further investigate the role of learning in the comparative study, Fig. 7 illustrates how the users’ performance improves as the users actively progress their task, regardless of the order of the conditions. In particular, Fig. 7A and B showcase that participants become faster and more accurate in localizing the tumor as they practice, showing strong adaptation to the different proposed interfaces. Moreover, the error from the selected location to the target is shown to decrease as the task proceeds both in the case of correct localization (Fig. 7C) and considering all the recorded trials (Fig. 7D). These results indicate how the introduction of a novel device such as the soft bilateral physical twin requires learning and adaptation, thus demonstrating that the results reported in Fig. 6 are just an initial characterization of the proposed interface, and the performance is bound to increase drastically as users train and learn how to correctly interpret and exploit ass the soft physical twin’s features. User-specific metric results are provided in Fig. S6.

## Discussion

In this paper, we propose the first reported case of a soft bilateral physical twin as a leader in a teleoperated system for remote palpation. The soft bilateral physical twin allows the human user to receive distributed tactile haptic feedback and to control the follower’s motion just by performing palpation onto the physical twin as if it were in person. For a complete characterization, such an interface, and all its sub-components, have been compared to traditional interfaces used in remote palpation such as a keyboard for the control and a visual pressure map for the feedback of the tactile data.

It has been shown that the physical twin is able to deliver feedback and control the follower’s motion. A closer look at individual trials reveals that the achieved trajectories are similar when compared to traditional control, with visible but limited unwanted lateral motion when pressing down toward the phantom. The proposed device is also able to explore a similar area as the traditional interface. Noticeably, the combination of soft control and visual feedback obtains an overall 5.1% higher accuracy than traditional methods in localizing the nodule. Such a result can be explained by the soft control resulting in faster trajectories than the keyboard when comparing conditions with the same feedback. However, when using tactile haptic feedback, participants show a decrease in accuracy, possibly due to the lower spatial resolution. Moreover, using both visual and tactile haptic feedback does not improve the performance, but rather overwhelms the participants, resulting in even lower accuracy than either one of the two feedbacks individually. Noticeably, the introduction of soft control results in a less invasive procedure, with 41% less force exchanged with the phantom. Nevertheless, the physical twin increases the required time to complete the task, with the exception of keyboard control and both feedbacks.

We believe that the proposed technology has the potential to be used in conjunction with or as an alternative to more traditional teleoperation interfaces. Concerning the achieved kinematics of the follower, it matches their speed and exploration capabilities, while resulting in a more gentle interaction with the remote environment. We acknowledge that the soft physical twin has been proven to require more time per trial and the tactile haptic feedback does not result in the same accuracy as the visual map. While the latter issue can be traced back to the lower spatial resolution of the physical twin (5 chambers) with respect to the visual maps (16 taxels), we believe the former to be the result of the participants’ learning process. The bilateral soft physical twin, unlike a keyboard and a screen, is an innovative new interface never before used by the participants, thus their performance can significantly increase far beyond what has been shown in this work in the long term. To support this claim, the learning curves of accuracy, error, and time required after first contact with the nodule show a great improvement in the participant’s performance over time, regardless of the testing conditions’ order.

On one hand, we endorse further studies on the role of training and how it would affect users’ performance in the long term. On the other, we acknowledge that the controlled setting that has been implemented in this study is far from a real-life scenario, thus the ecological validity of such a technology must be further investigated. Moreover, the current size of the setup is not suitable for being used in an already crowded medical setting. Future works on the miniaturization of the actuation technology can allow having a much higher spatial resolution of the tactile haptic feedback, thus resulting in a more informative feedback signal and better accuracy, as well as increasing the ecological validity of the device itself. Overall, we have proposed a soft, compact, and cheap device that can be used as a leader in a teleoperated architecture. Despite the required adaptation by the human participants, the bilateral soft physical twin has been shown to achieve similar kinematics level performance as traditional interfaces, while resulting in a much less invasive procedure and showing potential to increase diagnostic accuracy. This can pave the way for a new generation of leader interfaces with a larger focus on soft object interaction and preservation of the sense of touch.

## Materials and methods

### Participants

12 volunteering participants between 23 and 34 years of age ($25.8 \pm 2.7$ years), of which 6 males and 6 females, have been selected for the experiments. All participants self-identified as right-hand dominant. All participants elected to use their right hand to interact with the physical twin when they were not required to use the keyboard and their left hand when it was. All participants were either post-graduate students or members of staff of the University of Cambridge and the University of Oxford. Among the participants, 6 have an engineering background, and 6 have a life science background. All participants had no previous experience in the use of teleoperated systems and/or robotic-assisted navigation. No participant is a practicing medical doctor. It has been elected to perform the study on people with no experience in in-person clinical palpation, as previously done by similar studies on the topic^40^, in order to achieve a wider generalization of the potentialities showcased by the introduction of physical twins and to minimize biases caused by the uncanny valley between in-person and remote palpation^41^. All participants have either normal or corrected to normal vision. Written informed consent was obtained from all participants.

### Experimental design

Before the start of the experimental session, both control methods and feedback methods are explained to every participant, as well as the aim of the task. Then, a first trial is run using soft control and both visual and tactile haptic feedback in order to familiarize with the physical twin. In the experimental session, every participant undergoes all the possible permutations of control and feedback methods, for a total of 6 testing conditions: keyboard with visual feedback, keyboard with haptic feedback, keyboard with both visual and haptic feedback, soft control with visual feedback, soft control with haptic feedback, soft control with both visual and haptic feedback. The order in which every participant undergoes these conditions is randomized, so as to mitigate the effect of the learning curve on the results. For every condition, the participant undergoes 8 trials. In each trial, the user is required to remotely localize a hard nodule within a silicone phantom, as described in section “Task”. As a result, every participant undergoes a total of 48 trials. Every trial has a time limit of 90 s, and the participants rest for 300 s between conditions. Overall, the experimental session can last up to 97 mins, or closer to 2 h when considering the initial explanation and test run.

All methods were performed following the relevant guidelines and regulations, in accordance with the Declaration of Helsinki. The experiment protocol was approved by the ethics committee of the Department of Engineering, University of Cambridge, UK (Soft interface for bilateral teleoperation, application number 187).

### Task

The task consists of nodule localization through remote palpation: participants are required to correctly localize the hard nodule present in the silicon phantom as rapidly as possible. In each trial, the nodule is randomly placed by an operator in one of four possible locations so that every location is selected twice. This is achieved by rotating the phantom by 0, 90, 180, or 270° . Note that the participants do not know that the nodule can only be in four discrete locations, but are instructed that it can be anywhere in the phantom. The trial finishes either when the time limit of 90 s is reached or when the participants make their guesses about the nodule’s location. Participants can decide to end the trial at any time and their last end-effector location is recorded as their guess. The trial time starts only when the participants start moving the follower. Fig. S2 illustrates the flowchart followed by the software during the task.

### Apparatus

#### Manufacturing

The soft physical twin is obtained by multi-silicone casting, as shown in Fig. 3. This allows the directional control of the interface’s deformation^42^ and minimized inter-chamber cross-noise. All molds are made of PLA (*RS PRO PLA 3D Printer Filament, RS Group plc*) and have been obtained by 3D printing (*Ender-3 S1 Plus 3D Printer, Creality*). First, Dragon Skin 30 (*Smooth-On, Inc.*) is manually mixed in a 1:1 monomer and hardener ratio for 2 mins and placed in the vacuum pump for 20 mins to remove air bubbles. Then, the mold for the inner core is assembled, and the mixed silicone is cast into it, with excess material removed by tape casting. After 24 h, the external part of the mold is removed and replaced with the mold of the final product, composed of the external wall and the sensing chambers’ negatives. Because the new external wall is 3 mm higher than the actuated chambers’ negatives, vinyl tape (*Vynil Tape, RS Group plc*) is placed on top of the inner core, and the Dragon Skin 30 is manually covered in a layer of silicone glue (*Sil-poxy, Smooth-On, Inc.*) to ensure a strong interface between the two silicones able to withstand the actuation pressure. Then, Ecoflex 00-10 (*Smooth-On, Inc.*) is manually mixed in a 1:1 monomer and hardener ratio for 2 mins and placed in the vacuum pump for 20 mins to remove air bubbles. After the vinyl tape has been removed, the Ecoflex 00-10 is cast and left curing for 24 h. Similarly, for the physical twin’s base, Dragon Skin 30 is manually mixed in a 1:1 monomer and hardener ratio for 2 mins and placed in the vacuum pump for 20 mins to remove air bubbles. Then, the mold for the base is assembled, and the mixed silicone is cast into it, with excess material removed by tape casting. The role of the base is to provide easy access to the inner chambers, used for actuation. Further details about the molds used in silicone casting are provided in Fig. S1. The base and the main body of the physical twin are then manually connected using silicone glue, and all the chambers are connected to either the pressure sensors or the proportional valves using silicone tubes (*SenTech, Inc.*) and silicone glue to ensure air-tight connections.

#### Leader

As can be seen in Fig. 2A, the leader is composed of a client, a screen, a keyboard, and all the equipment needed to control and monitor the physical twin. The client’s role is to communicate with the server, sending the input commands and receiving the data collected from the camera and the tactile sensor. The communication between client and server has been developed ad hoc through TCP/IP via sockets and it is asynchronous so that the server can run at higher frequencies and better control the follower. The client also provides a GUI to the user through the screen. The GUI shows the real-time camera feed, and the tactile visual map when required by the testing condition, as well as informing the user of the remaining experiment time. The physical twin is entirely controlled using an Arduino (*Arduino Mega2560, Arduino*). The pressure inside the sensing chambers is recorded using barometric sensors (*40PC100G2A, Honeywell*), read by Arduino, and transferred to the client through serial communication. The actuated chambers are controlled using proportional pressure valves (*VEAB-B-26-D12-F-A4-1R1 8046266, Festo*). To deliver the analog output needed to control the valves, a multiplexer (*TCA9548A, SparkFun*) and a set of DACs (*MCP4725, SparkFun*) controlled by Arduino through I2C to achieve the desired chamber pressure, set by the client vis serial communication. Because of the presence of Arduino, the leader operates at 50 Hz.

#### Follower

The follower is composed of a server, the robotic arm (*UR5, Universal Robots*), the camera, and the tactile sensor with all the equipment needed to transfer its data to the server. The tactile sensor is a 16-taxels 3 DoF force sensor (*uSkin 4*$\times$*4, Xela Robotics*). Because of the dynamics of palpation, we solely use the normal force values in this study, as previously done in literature^35,37,39^. The recorded signal is communicated from the sensor to an IHB (*CySkin*) through I2C. A CAN-USB converter (*CAN-USB/2, ESD Electronics gmbh*) is then used to deliver the CAN communication to the server. The server also receives data asynchronously from the camera and controls the motion of the follower through the UR5 RTDE protocol. The follower’s workspace has been limited to the location of the silicone phantom and a maximum indentation of the phantom itself of $1\;{\rm cm}$, to avoid the possibility of triggering the safety mode of the UR5 during experimental trials. Because both the communication with the client and the camera feed are asynchronous, the follower is able to operate above 100 Hz.

#### Silicone phantom

In order to simulate the patient’s abdomen, a soft silicone phantom with a hard nodule is made. First, Ecoflex 00-20 (*Smooth-On, Inc.*) is manually mixed in a 1:1 monomer and hardener ratio for 2 mins and placed in the vacuum pump for 20 mins to remove air bubbles. Then, it is cast in a PLA 3D-printed mold. After 24 h, a PLA sphere with a 5 mm diameter is manually placed on top of the cured silicone. Then, more Ecoflex 00-20 is manually mixed in a 1:1 monomer and hardener ratio for 2 mins, while adding silicone pigment (*Smooth-On, Inc.*) to hide the location of the nodule, and placed in the vacuum pump for 20 mins to remove air bubbles. Lastly, the colored Ecoflex 00-20 is cast so as to entirely cover the PLA nodule and left curing for 24 h. The result is a 25 *mm* thick $100\times 100\;mm^2$ square phantom with threaded corners. The different nodule locations for the tests are obtained using a rotating platform on which the phantom is placed.

#### Visual tactile map

The visual tactile map is obtained directly from the data of the tactile sensors. It consists of 16 portions, everyone corresponding to the normal force recorded by each taxel, in the same spatial distribution as the taxels themselves on the tactile sensor. Every taxel is then individually scaled between minimum and maximum in order to be mapped to a blue scale, with white corresponding to maximum and blue to minimum, as follows:

1 $$\begin{aligned} Tx_i = \frac{V_i}{V^{max}_i} \end{aligned}$$

where $Tx_i$ is the normalized taxel $i\textrm{th}$ value, $V_i$ is the respective taxel raw value, and $V^{max}_i$ is the maximum value registered by such a taxel.

#### Tactile haptic feedback

The actuated chambers’ pressure is controlled by the taxel values. To adapt the 16 taxels to the 5 actuated chambers, the taxel values have been arranged as follows:

2 $$\begin{aligned} {\left\{ \begin{array}{ll} F_1=\alpha \frac{Tx_6+Tx_7+Tx_{10}+Tx_{11}}{4}\\ F_2=\alpha \frac{Tx_1/2+Tx_5+Tx_9+Tx_{13}/2}{3}\\ F_3=\alpha \frac{Tx_4/2+Tx_8+Tx_{12}+Tx_{16}/2}{3}\\ F_4=\alpha \frac{Tx_1/2+Tx_2+Tx_3+Tx_4/2}{3}\\ F_5=\alpha \frac{Tx_{13}/2+Tx_{14}+Tx_{15}+Tx_{16}/2}{3}\end{array}\right. } \end{aligned}$$

where *F* indicates the driving voltage to the actuated chambers used to deliver the tactile haptic feedback: $F_1$ is the central chamber, $F_2$ is the left one, $F_3$ is the one toward the user, $F_4$ is the right one, and $F_5$ is the one away from the user. *Tx* represents the normalized taxels, numbered from top left to bottom right with respect to their position on the sensor. $\alpha$ adapts the normalized taxel values to the chamber driving voltage. For the participants’ studies, $\alpha$ = 1.95. This value has been selected empirically as higher values would result in the mechanical failure of the physical twin.

#### Soft control

From the pressure recorded in the sensing cavities, the follower velocity is updated as follows:

3 $$\begin{aligned} {\left\{ \begin{array}{ll} v_x= v_0(u(P_1-\theta _0)+u(P_3-\theta _0))\\ v_y=v_0(u(P_4-\theta _0)+u(P_2-\theta _0))\\ v_z=v_0\Pi ^{4}_{i=1}u(P_i-\theta _1) \end{array}\right. } \end{aligned}$$

where $v_i$ is the follower’s velocity along the *x*, *y*, and *z* axes and *P* indicates the pressure recorded inside the sensing chambers: $P_1$ is the left one, $P_2$ is the one toward the user, $P_3$ is the right one, and $P_4$ is the one away from the user. $u(\cdot )$ is the step function and $v_0$ is a constant speed selected to be $20\;\textrm{mm}/\textrm{s}$. For the participants’ study, the threshold values $\theta _0$ and $\theta _1$ have been selected as 0.09 and 0.01, respectively, based on previous studies of such technology^38^.

#### Keyboard control

The keyboard control has been implemented as shown in previous studies^39^: *A* and *D* are positive and negative velocity along the *x* axis, *W* and *S* are positive and negative velocity along the *y* axis, and *Space* and *Alt* are positive and negative velocity along *z*, respectively. While pressing a key, the robotic arm’s end effector moves toward the desired direction at a constant speed $v_0=20\;\textrm{mm}/\textrm{s}$.

### Data collection

During each trial, the position of the follower’s end-effector, the relative time with respect to the trial’s start, the taxel values of the tactile sensor, and the inner pressure of both sensing and actuation chambers have been recorded with a frequency of 50 Hz from the start of the trial to its end, determined either by the user upon the localization of the nodule or by the reaching of the maximum trial time of 90 s.

### Data processing

#### Trial’s metrics

Figure 5 shows raw data from individual trials. Conversely, Figs. 6 and 7 are obtained by processing all trials of all participants and extracting for every trial the five metrics of interest. In Fig. 6, data are displayed as a function of the testing control and feedback conditions, whereas in Fig. 7 they are displayed as a function of the order in which the conditions have been executed. For every single trial, the metrics of interest have been computed as follows:

**Accuracy:** For every tested condition, the accuracy is computed as the ratio between the trials in which the nodule was within the area covered by the final position of the tactile sensor and the overall number of trials.

**Time:** For every trial, time is recorded as the total time from the beginning of the trial to either the user’s guess or the $90\;s$ time limit.

**Area:** For every trial, the area is computed as the ratio between the explored area and the total phantom’s area. Any portion of the area is considered explored if contact is made between the tactile sensor and the phantom itself. Contact is detected by an increase of 2% or higher of the recorded value of any taxel.

**Force:** For every trial, the average force exchanged with the phantom is computed as follows:

4 $$\begin{aligned} Force = \frac{\int _{t=0}^{T} \sum _{i=0}^{16}V_i \; dt}{ T} \end{aligned}$$

where *T* is the trial’s time, and $V_i$ is the $i^{th}$ taxel.

**Speed:** For every trial, the average speed is computed as follows:

5 $$\begin{aligned} Speeed = \frac{ \int _{t=0}^{T} (|x'(t)|+|y'(t)|+|z'(t)|) \; dt}{ T} \end{aligned}$$

where *T* is the trial’s time, $x,\; y$ and $z$ are the 3 Cartesian axes, $|\cdot |$ is the absolute value operation, and $'$ indicates derivative with respect to time.

**Time after contact:** For every trial, the time after contact is computed as the time from the first time contact is made with the nodule to the trial’s end time.

**Error:** The error is computed as the Euclidean distance from the user’s guess to the real location of the nodule. Fig. 7C is obtained by using only the trials in which the nodule’s location was correctly localized, whereas  7D is obtained using all trials.

#### Radar chart

For a compact representation of the main differences among interfaces, Fig. 6B illustrates a radar chart that compares all interfaces across the 5 selected metrics: accuracy, time, area, force, and speed. The chart reports the average among all participants and trials as a function of the control and feedback conditions. Accuracy, speed, and area are computed as reported in section “Trial’s metrics”. Conversely, in order to maintain a coherent way of reading the chart, force and time have been computed differently: force is computed as 1/force, and time is computed as 90 s−time. All 5 metrics have then been re-scaled with respect to the maximum among all tested conditions, in order to highlight differences among them.

#### Statistical tests

In order to investigate the statistical relevance of the obtained results, both one-way ANOVA and paired t-tests have been implemented. Moreover, a Shapiro-Wilk test has been performed to investigate the normality of the tested data. To ensure the required independence of data points, the datasets for the statistical tests have been obtained by computing the mean of every metric for each participant and testing condition, as follows:

6 $$\begin{aligned} m^{j,k} = \frac{ \sum _{i=0}^{8} m^{j,k}_i}{8} \end{aligned}$$

where *m* indicates the metric, *j* the participant, *k* the testing conditions, and *i* the trial number, up to 8, for the given participant and conditions. The resulting dataset is composed of 12 independent data points for each condition, as they belong to the 12 different participants. Such datasets have been used to perform the aforementioned tests, and the results are reported in Table 1, S1, and S2–S6 for the ANOVA, Shapiro-Wilk test and paired t-test, respectively.

### Supplementary Information

Supplementary Information 1.

Supplementary Information 2.

## Data Availability

All the data needed to evaluate the study are in the main text or in the Supplementary Materials. The datasets generated and analyzed during the current study and codes are available upon request to the corresponding author Leone Costi.

## References

[1] Bilal M (2017). The Clinical anatomy of the physical examination of the abdomen: A comprehensive review. Clin. Anatomy.

[2] Alpert JS (2019). How accurate are the findings noted during a physical examination?: Will physicians stop performing physical examinations? (Part 2). Am. J. Med..

[3] Palacio-Torralba J, Reuben RL, Chen Y (2020). A novel palpation-based method for tumor nodule quantification in soft tissue-computational framework and experimental validation. Med. Biol. Eng. Comput..

[4] Konstantinova J, Cotugno G, Dasgupta P, Althoefer K, Nanayakkara T (2017). Palpation force modulation strategies to identify hard regions in soft tissue organs. PLoS ONE.

[5] Scheffler RM, Liu JX, Kinfu Y, Dal Poz MR (2008). Forecasting the global shortage of physicians: An economic- and needs-based approach. Bull. World Health Org..

[6] Hokayem PF, Spong MW (2006). Bilateral teleoperation: An historical survey. Automatica.

[7] Mehrdad S, Liu F, Pham MT, Lelevé A, Farokh-Atashzar S (2021). Review of advanced medical telerobots. Appl. Sci. (Switzerl.).

[8] Troccaz J, Dagnino G, Yang GZ (2019). Frontiers of medical robotics: From concept to systems to clinical translation. Annu. Rev. Biomed. Eng..

[9] Chen Y (2020). Review of surgical robotic systems for keyhole and endoscopic procedures: State of the art and perspectives. Front. Med..

[10] Kim SY, Kyung KU, Park J, Kwon DS (2007). Real-time area-based haptic rendering and the augmented tactile display device for a palpation simulator. Adv. Robot..

[11] Herzig N, Maiolino P, Iida F, Nanayakkara T (2018). A variable stiffness robotic probe for soft tissue palpation. IEEE Robot. Autom. Lett..

[12] Takács Á, Rudas IJ, Haidegger T (2019). The other end of human-robot interaction: Models for safe and efficient tool-tissue interactions. Hum.-Robot Interact..

[13] Scimeca L (2022). Action augmentation of tactile perception for soft-body palpation. Soft Robot..

[14] Intuitive surgical annual report 2018. (2018).

[15] Alletti SG (2018). The senhance™ surgical robotic system (senhance) for total hysterectomy in obese patients: A pilot study. J. Robot. Surg..

[16] Lang S (2017). A european multicenter study evaluating the flex robotic system in transoral robotic surgery. The Laryngoscope.

[17] Kelkar D, Borse MA, Godbole GP, Kurlekar U, Slack M (2021). Interim safety analysis of the first-in-human clinical trial of the versius surgical system, a new robot-assisted device for use in minimal access surgery. Surg. Endosc..

[18] Panëels S, Roberts JC (2010). Review of designs for haptic data visualization. IEEE Trans. Hapt..

[19] Culbertson H, Schorr SB, Okamura AM (2018). Haptics: The present and future of artificial touch sensation. Annu. Rev. Control Robot. Auton. Syst..

[20] Amirabdollahian F (2018). Prevalence of haptic feedback in robot-mediated surgery: A systematic review of literature. J. Robot. Surg..

[21] El Rassi I, El Rassi JM (2020). A review of haptic feedback in tele-operated robotic surgery. J. Med. Eng. Technol..

[22] Salud LH, Pugh CM (2011). Use of sensor technology to explore the science of touch. Stud. Health Technol. Inf..

[23] Auvray, M. & Duriez, C. *Haptics: Neuroscience, Devices, Modeling, and Applications* vol. 8618 (Springer, 2014).

[24] Racat M, Capelli S (2020). When Interfaces Make It Real.

[25] Li, M., Luo, S. & Xu, G. A tactile sensing and feedback system for tumor localization. In *2016 13th International Conference on Ubiquitous Robots and Ambient Intelligence, URAI 2016* 259–262. 10.1109/URAI.2016.7625751 (2016).

[26] Li M (2017). Evaluation of stiffness feedback for hard nodule identification on a phantom silicone model. PLoS ONE.

[27] Kim, J., Ahn, B., Kim, Y. & Kim, J. Inclusion detection with haptic-palpation system for medical telediagnosis. In *Proceedings of the 31st Annual International Conference of the IEEE Engineering in Medicine and Biology Society: Engineering the Future of Biomedicine, EMBC 2009* 4595–4598. 10.1109/IEMBS.2009.5332767 (2009). 10.1109/IEMBS.2009.5332767 19963847

[28] Coles TR, John NW, Gould D, Caldwell DG (2011). Integrating haptics with augmented reality in a femoral palpation and needle insertion training simulation. IEEE Trans. Hapt..

[29] Li M (2015). Using visual cues to enhance haptic feedback for palpation on virtual model of soft tissue. Med. Biol. Eng. Comput..

[30] Tzemanaki A, Al GA, Melhuish C, Dogramadzi S (2018). Design of a wearable fingertip haptic device for remote palpation: Characterisation and interface with a virtual environment. Front. Robot. AI.

[31] Talasaz, A. & Patel, R. V. Remote palpation to localize tumors in robot-assisted minimally invasive approach. In *Proceedings - IEEE International Conference on Robotics and Automation* 3719–3724. 10.1109/ICRA.2012.6224649 (2012).

[32] Feller RL, Lau CK, Wagner CR, Perrin DP, Howe RD (2004). The effect of force feedback on remote palpation. Proc. IEEE Int. Conf. Robot. Autom..

[33] Roke C, Melhuish C, Pipe T, Drury D, Chorley C (2012). Lump localisation through a deformation-based tactile feedback system using a biologically inspired finger sensor. Robot. Auton. Syst..

[34] Costi, L., Maiolino, P. & Iida, F. Soft Morphing Interface for Tactile Feedback in Remote Palpation. In *2022 IEEE 5th International Conference on Soft Robotics (RoboSoft)* 01–06. 10.1109/RoboSoft54090.2022.9762173 (IEEE, 2022).

[35] Hernandez-ossa, K. A., Leal-junior, A. G., Frizera-neto, A. & Bastos, T. Haptic Feedback for Remote Clinical Palpation Examination (2019).

[36] Dargahi J, Xie WF, Ji P (2008). An experimental teletaction system for sensing and teleperception of human pulse. Mechatronics.

[37] Li W, Shi L, Deng H, Zhou Z (2014). Investigation on friction trauma of small intestine in vivo under reciprocal sliding conditions. Tribol. Lett..

[38] Costi, L., Lalitharatne, T. D. & Iida, F. Soft control interface for highly dexterous unilateral remote palpation. In *2022 9th IEEE RAS/EMBS International Conference for Biomedical Robotics and Biomechatronics (BioRob)* 1–8 (IEEE, 2022).

[39] Costi L (2021). Comparative analysis of model-based predictive shared control for delayed operation in object reaching and recognition tasks with tactile sensing. Front. Robot. AI.

[40] Lalitharatne TD (2022). Face mediated human-robot interaction for remote medical examination. Sci. Rep..

[41] Costi, L. & Iida, F. How to define the correct guidelines for enhanced telepresence and task embodiment in remote palpation. In *IOP Conference Series: Materials Science and Engineering, vol. 1292* 012024 (IOP Publishing, 2023).

[42] Costi, L., Maiolino, P. & Iida, F. Soft morphing interface for tactile feedback in remote palpation. In *2022 IEEE 5th International Conference on Soft Robotics (RoboSoft)* 01–06 (IEEE, 2022).

