# Supplementary material for: Multi-silicone bilateral soft physical twin as an alternative to traditional user interfaces for remote palpation: a comparative study
Source: Sci Rep. 2023 Dec 27;13:23014. doi: 10.1038/s41598-023-50329-4 (PMC10754944; doi:10.1038/s41598-023-50329-4)
Supplement: Supplementary file 1 — Supplementary Information 1. [file 41598_2023_50329_MOESM1_ESM.pdf]

Supplementary Material

Movie S1. A video taken during different trials showcasing the differences in the various interfaces both in terms of control and feedback.

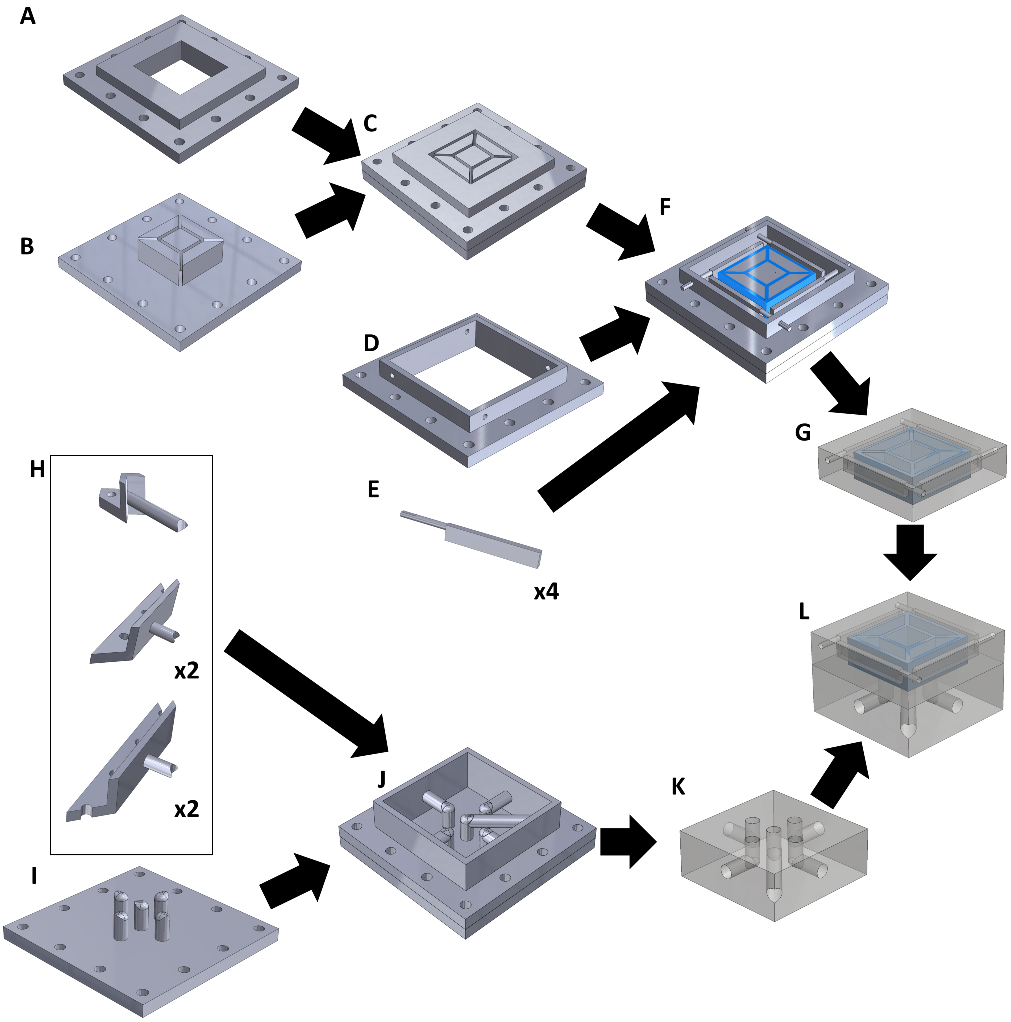

Figure S1. Detailed mold assembly and physical twin fabrication process. (A) external and (B) internal components of the (C) Dragon Skin 30 core's mold. (D) external component and (E) chambers' negatives to assemble the (F) Ecoflex 00-10 mold to fabricate the (G) main body of the physical twin. (H) External and (I) internal components of the (J) Dragon Skin 30 base mold used to achieve the (K) base of the physical twin. The main body and the base are then manually assembled to create the physical twin. The PLA molds are gray, whereas the Silicone is transparent. To allow better visibility of the internal design, the Dragon Skin 30 core within the main body is highlighted in blue.

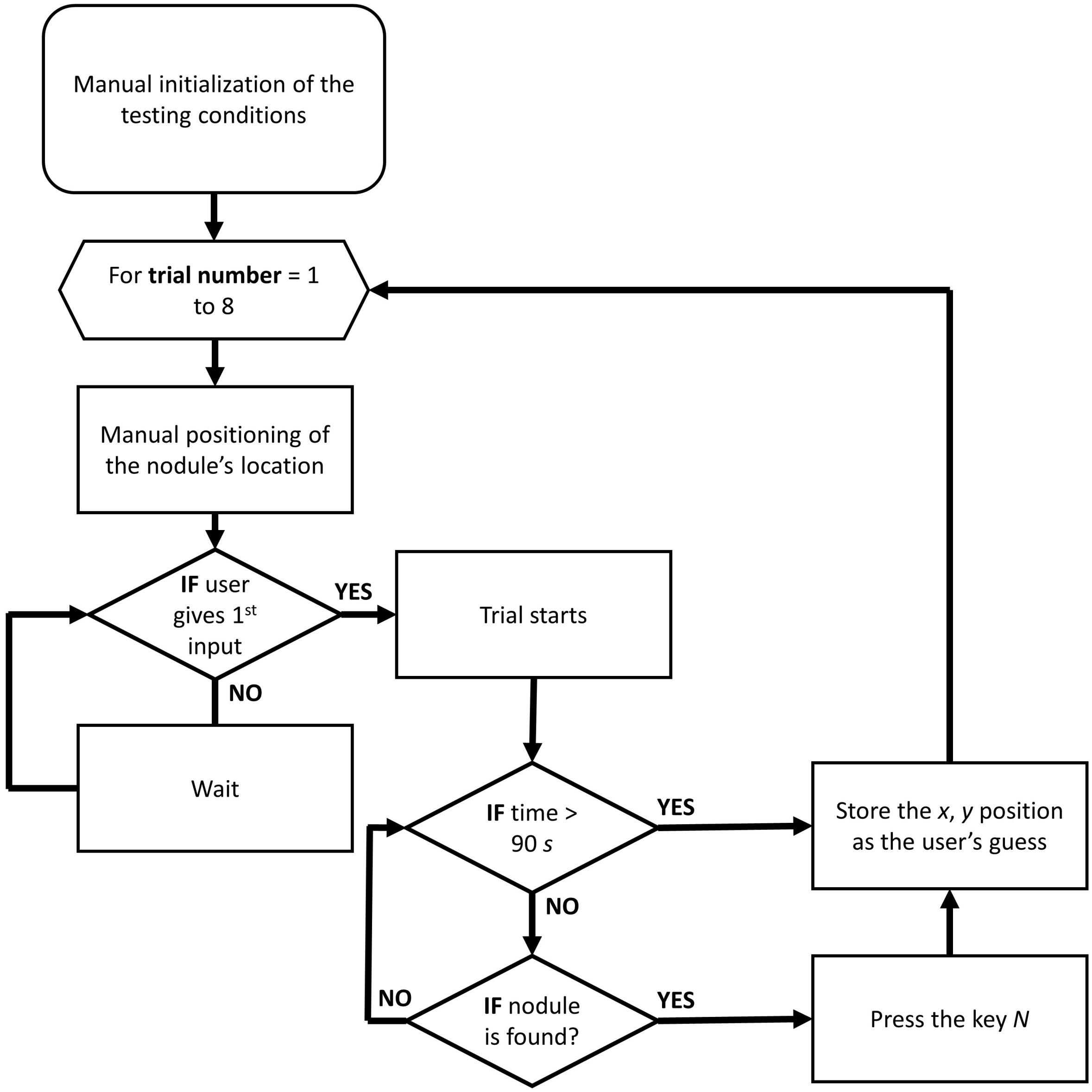

Figure S2. Flow chart of the experimental sequence used during each of the 6 different testing conditions. The testing conditions are as follows: keyboard control and visual feedback, keyboard control and haptic feedback, keyboard control and both feedbacks, soft control and visual feedback, soft control and haptic feedback, soft control, and both feedbacks.

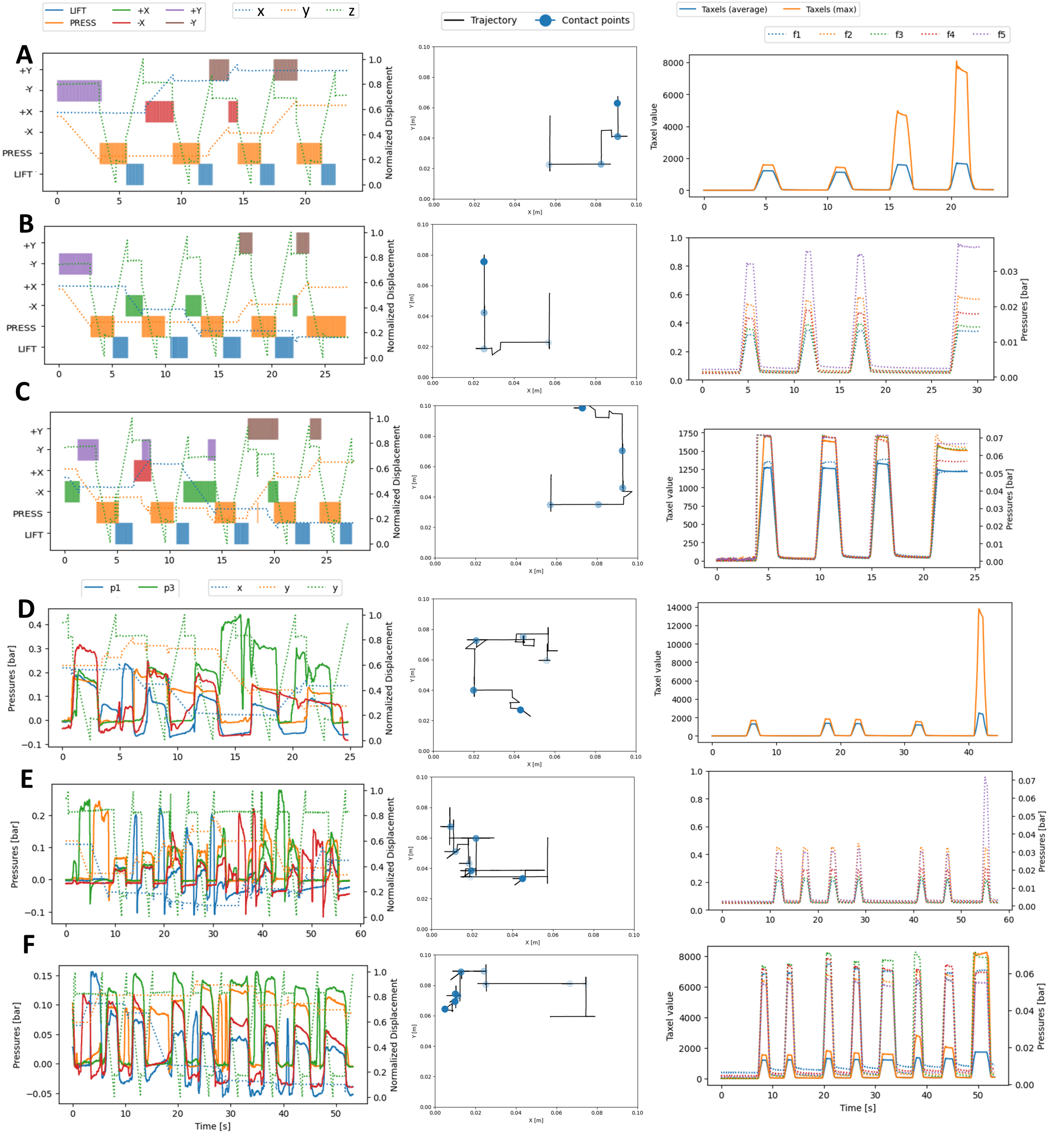

Figure S3. Examples of user inputs achieved trajectory, and feedback signals in the case of (A) keyboard control and visual feedback, (B) keyboard control and haptic feedback, (C) keyboard control and both feedbacks, (D) soft control and visual feedback, (E) soft control and haptic feedback, (F) soft control and both feedbacks. In all plots, solid lines refer to the left y axis, and dashed lines refer to the right y axis. In the trajectory plots in the central column, the intensity of the marker represents the time: lighter points have been palpated at the beginning of the trial, whereas darker ones have been palpated toward the end.

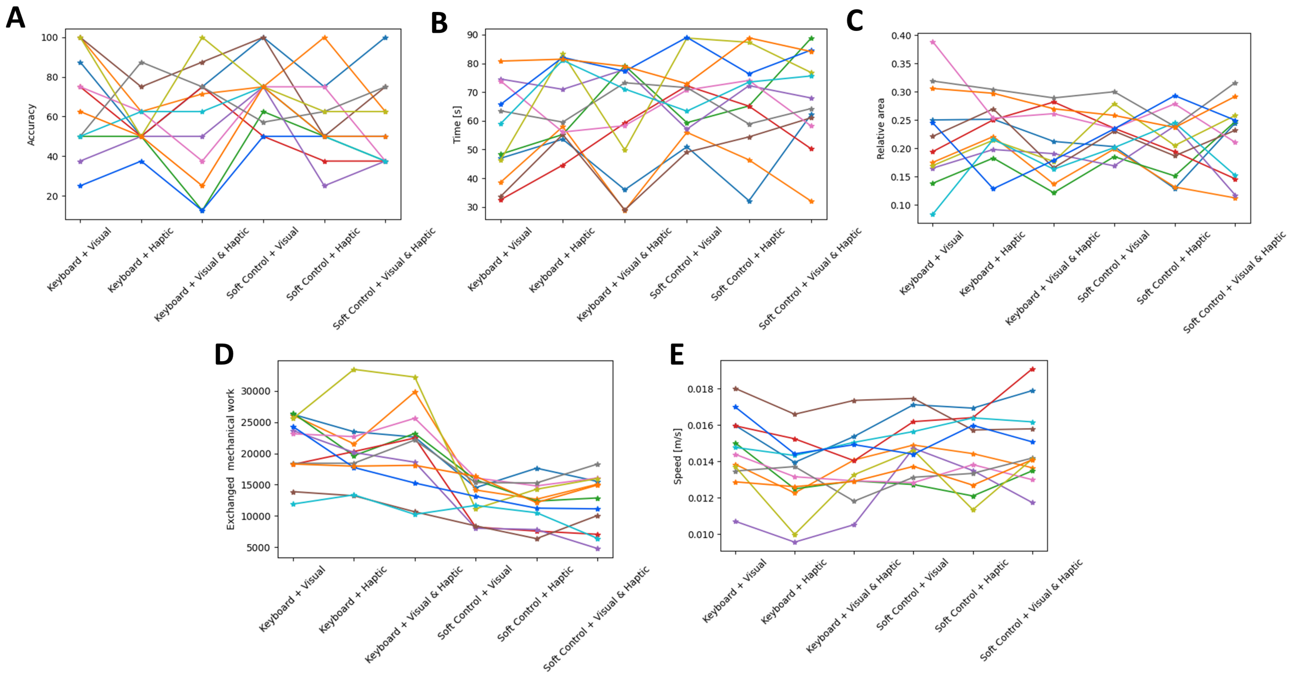

Figure S4. User-specific results divided into the 5 selected metrics: (A) accuracy, (B) time, (C) explored area, (D) exchanged mechanical work, and (E) average speed. Every data point is the average of a single user over the 8 trials performed under a given condition.

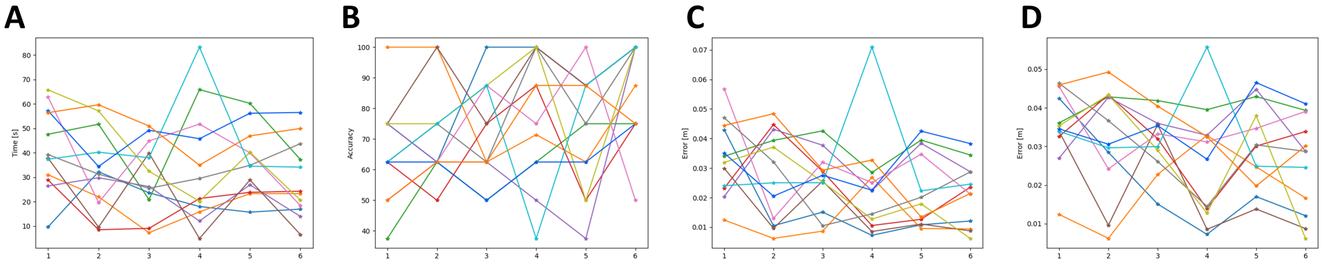

Figure S5. User-specific learning curves in the case of (A) time needed to localize the nodule after the first contact was made, (B) accuracy, (C) error when the tumor was correctly localized, and (D) general error.

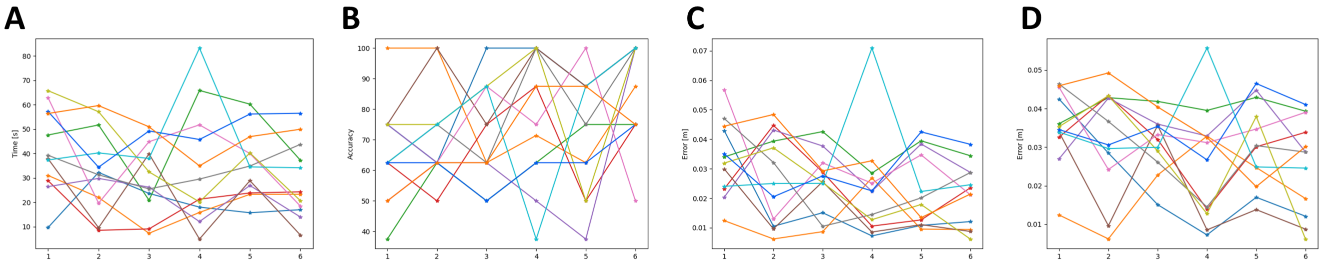

Figure S6. User-specific learning curves in the case of (A) time needed to localize the nodule after the first contact was made, (B) accuracy, (C) error when the tumor was correctly localized, and (D) general error.

Table S1. Shapiro-Wilk test results.

CalcW	Keyboard + Visual	Keyboard + Haptic	Keyboard + Visual & Haptic	Soft Control + Visual	Soft Control + Haptic	Soft Control + Visual & Haptic
Accuracy	0.855	0.864	0.910	0.737	0.924	0.933
Time	0.939	0.859	0.849	0.923	0.956	0.949
Area	0.974	0.964	0.913	0.961	0.942	0.922
Force	0.872	0.879	0.959	0.879	0.967	0.915
Speed	0.983	0.958	0.976	0.945	0.936	0.942
p-value	Keyboard + Visual	Keyboard + Haptic	Keyboard + Visual & Haptic	Soft Control + Visual	Soft Control + Haptic	Soft Control + Visual & Haptic
Accuracy	0.042	0.055	0.213	0.002	0.321	0.421
Time	0.481	0.048	0.035	0.316	0.730	0.818
Area	0.945	0.834	0.234	0.800	0.525	0.304
Force	0.068	0.084	0.773	0.084	0.875	0.246
Speed	0.993	0.762	0.964	0.560	0.450	0.537

Table S2. Accuracy paired t-test results. For each test, the condition reported in the first row and the one reported in the first column are the first and second inputs, respectively.

t-ratio	Keyboard + Visual	Keyboard + Haptic	Keyboard + Visual & Haptic	Soft Control + Visual	Soft Control + Haptic
Keyboard + Haptic	1.356				
Keyboard + Visual & Haptic	0.994	-0.622			
Soft Control + Visual	-1.894	-5.745	-2.733		
Soft Control + Haptic	1.400	-0.491	0.250	3.447	
Soft Control + Visual & Haptic	2.708	0.321	1.090	3.838	0.748

p-value	Keyboard + Visual	Keyboard + Haptic	Keyboard + Visual & Haptic	Soft Control + Visual	Soft Control + Haptic
Keyboard + Haptic	0.201				
Keyboard + Visual & Haptic	0.341	0.547			
Soft Control + Visual	0.085	<0.001	0.019		
Soft Control + Haptic	0.020	0.633	0.807	0.005	
Soft Control + Visual & Haptic	0.201	0.754	0.299	0.003	0.470

Table S3. Time paired t-test results. For each test, the condition reported in the first row and the one reported in the first column are the first and second inputs, respectively.

t-ratio	Keyboard + Visual	Keyboard + Haptic	Keyboard + Visual & Haptic	Soft Control + Visual	Soft Control + Haptic
Keyboard + Haptic	-2.293				
Keyboard + Visual & Haptic	-1.099	0.969			
Soft Control + Visual	-2.223	-0.446	-1.293		
Soft Control + Haptic	-2.396	-0.319	-1.390	0.163	
Soft Control + Visual & Haptic	-2.423	-0.528	-1.751	-0.086	-0.223
p-value	Keyboard + Visual	Keyboard + Haptic	Keyboard + Visual & Haptic	Soft Control + Visual	Soft Control + Haptic
Keyboard + Haptic	0.043				
Keyboard + Visual & Haptic	0.295	0.353			
Soft Control + Visual	0.048	0.664	0.222		
Soft Control + Haptic	0.036	0.755	0.192	0.873	
Soft Control + Visual & Haptic	0.034	0.607	0.108	0.933	0.828

Table S4. Area paired t-test results. For each test, the condition reported in the first row and the one reported in the first column are the first and second inputs, respectively.

t-ratio	Keyboard + Visual	Keyboard + Haptic	Keyboard + Visual & Haptic	Soft Control + Visual	Soft Control + Haptic
Keyboard + Haptic	-0.510				
Keyboard + Visual & Haptic	0.992	2.167			
Soft Control + Visual	-0.295	0.363	-1.739		
Soft Control + Haptic	0.439	0.952	-0.381	1.048	
Soft Control + Visual & Haptic	0.292	-0.510	0.862	0.887	-0.176

p-value	Keyboard + Visual	Keyboard + Haptic	Keyboard + Visual & Haptic	Soft Control + Visual	Soft Control + Haptic
Keyboard + Haptic	0.620				
Keyboard + Visual & Haptic	0.343	0.053			
Soft Control + Visual	0.774	0.723	0.110		
Soft Control + Haptic	0.669	0.362	0.711	0.317	
Soft Control + Visual & Haptic	0.776	0.407	0.620	0.394	0.864

Table S5. Force paired t-test results. For each test, the condition reported in the first row and the one reported in the first column are the first and second inputs, respectively.

t-ratio	Keyboard + Visual	Keyboard + Haptic	Keyboard + Visual & Haptic	Soft Control + Visual	Soft Control + Haptic
Keyboard + Haptic	1.013				
Keyboard + Visual & Haptic	0.329	-0.758			
Soft Control + Visual	5.980	4.377	4.312		
Soft Control + Haptic	7.373	6.277	5.511	1.318	
Soft Control + Visual & Haptic	5.961	5.276	5.379	0.463	-0.635

p-value	Keyboard + Visual	Keyboard + Haptic	Keyboard + Visual & Haptic	Soft Control + Visual	Soft Control + Haptic
Keyboard + Haptic	0.333				
Keyboard + Visual & Haptic	0.749	0.465			
Soft Control + Visual	<0.001	0.001	0.001		
Soft Control + Haptic	<0.001	<0.001	<0.001	0.214	
Soft Control + Visual & Haptic	<0.001	<0.001	<0.001	0.652	0.539

Table S6. Speed paired t-test results. For each test, the condition reported in the first row and the one reported in the first column are the first and second inputs, respectively.

t-ratio	Keyboard + Visual	Keyboard + Haptic	Keyboard + Visual & Haptic	Soft Control + Visual	Soft Control + Haptic
Keyboard + Haptic	4.466				
Keyboard + Visual & Haptic	3.348	-1.474			
Soft Control + Visual	-0.278	-2.910	-2.744		
Soft Control + Haptic	0.520	-2.846	-1.421	1.048	
Soft Control + Visual & Haptic	-0.440	-3.562	-2.255	-0.160	-1.123
**p-value**	**Keyboard + Visual**	**Keyboard + Haptic**	**Keyboard + Visual & Haptic**	**Soft Control + Visual**	**Soft Control + Haptic**
Keyboard + Haptic	0.001				
Keyboard + Visual & Haptic	0.007	0.169			
Soft Control + Visual	0.787	0.014	0.019		
Soft Control + Haptic	0.613	0.016	0.183	0.317	
Soft Control + Visual & Haptic	0.668	0.004	0.045	0.876	0.286
